# Lightning injuries in a lightning city: a district hospital experience in Singapore

**DOI:** 10.1186/cc11091

**Published:** 2012-03-20

**Authors:** Y Mok, P Tan, R Jagadesan

**Affiliations:** 1 Changi General Hospital, Singapore

## Introduction

Tropical Singapore's meterological profile makes it one of the world's lightning capitals. This study aims to assess the profile of the at-risk patient, and possibly identify factors predicting the length of hospital stay in patients with lightning injuries over a period of 11 years.

## Methods

This is an 11-year retrospective study of patients who were admitted to Changi General Hospital, the only hospital located in eastern Singapore, from 2000 to 2011 with the diagnosis of lightning injuries.

## Results

There were a total of 27 subjects, with 25 (95.6%) males and two (7.4%) females in the sample. Their age ranged from 17 to 62 years; 63% of the subjects were between 20 and 40 years old. All except three subjects had no comorbidities, with the latter having only hypertension or hyperlipidemia. Most of the events occurred during two periods, March to April and October to December, which is consistent with previously observed seasonal peaks. The length of hospital stay ranged from 1 to 10 days for all patients, except one who stayed for 78 days and one who was transferred to another hospital. Six patients (22.2%) required admission to the ICU or high dependency. There were three mortalities, all found in asystole at the incident site and also suffered hypoxic ischemic encephalopathy (HIE). Seventeen (63%) events were occupation related with all occurring either at the airbase or open construction sites. Although there were reportedly six mechanisms of lightning injuries (direct strike, contact injury, side flash, ground current, upward streamer and blast injury) this study only established two types of mechanisms - direct strike and contact injury - amongst our patients. Clinical and biochemical parameters that were studied included cardiovascular morbidity, rhabdomyolysis, otologic injuries, burns, acute kidney injury and neurological complications. The small numbers limited a statistical analysis for any correlations between clinical factors and prognosis as well as hospital length of stay. Nevertheless, it is notable that all three deaths had asystole arrest at presentation, developed HIE, and a trend towards a higher serum creatinine on admission.

## Conclusion

The results of this study add to the small but increasing literature on lightning injuries and may serve to increase physician awareness in this medical niche.
